# Commentary: Ancient genomes show social and reproductive behavior of early Upper Paleolithic foragers

**DOI:** 10.3389/fpsyg.2017.02247

**Published:** 2017-12-19

**Authors:** Antonio Benítez-Burraco

**Affiliations:** Department of Spanish, Linguistics, and Theory of Literature (Linguistics), Universidad de Sevilla, Seville, Spain

**Keywords:** language evolution, language complexity, ancient genomes, hunter-gatherers, historical linguistics

Confident reconstructions of prehistoric languages are precluded by the widespread occurrence of borrowing resulting from language contact, which blurs the phylogenetic links between languages resulting from common ancestry. Because languages are cultural systems that can be learned and shifted (even when they are modeled by the cognitive faculty that enables us to acquire and use them), attempts to reconstruct language phylogenies based purely on the dynamics of human gene pools and particularly, on human genetic phylogenies have also failed to a great extent. As noted by Pakendorf (2014), although linguistic boundaries sometimes act as barriers to gene flow, genetic admixture frequently takes place irrespective of linguistic affiliations.

These circumstances explain why until now characterizations of prehistoric languages and models of language dynamics in the remote past have largely relied on linguistic evidence and theories. Typological surveys of present-day languages, as well as some celebrated depictions of how language is implemented in our brains (e.g., Chomskyan linguistics), have crystallized in a uniformitarian view of the nature of languages (Fromkin and Rodman, 1983; Dixon, 1997), according to which the languages spoken in the past were roughly equal to the languages we speak today in terms of overall complexity. Likewise, favorite theories in historical linguistics, like grammaticalization theory (which accounts for the emergence of functional words in the grammars of languages), have been used to circumvent the limits of linguistic reconstructions and to infer how languages (and language) might have been in remote prehistory (e.g., Heine and Kuteva, 2007). Nonetheless, the resulting picture, even if plausible, is difficult to prove, as no remains of the languages spoken at that times are available.

Recent research by anthropological and evolutionary linguists suggests that structural aspects of languages correlate with (and might depend on) environmental and social factors (Lupyan and Dale, 2016). In particular, small, close-knit human communities tend to speak languages with complex, opaque, and redundant morphologies, reduced semantic transparency, and limited syntactic devices, to the point that core aspects of human languages, like recursion, might be absent (Everett, 2005). On the contrary, increased cross-cultural exchanges, and ultimately, widespread language contact, are hypothesized to regularize morphological paradigms, augment semantic transparency, and trigger compositionality and more elaborated syntaxes (Bolender, 2007; Wray and Grace, 2007; Lupyan and Dale, 2010; Trudgill, 2011). Overall, this suggests that we might achieve a better understanding of the nature of prehistoric languages if we knew more about how social dynamics were in the past.

Until recently, our understanding of the socio-cultural milieu of Paleolithic humans was limited to what can be inferred from the archeological record. It seems, for instance, that Magdalenian humans (from ca. 15,000 years before present) lived in small bands with quite elaborated social systems and extensive long-distance contacts (Weniger, 1989; Schwendler, 2012). Not surprisingly, we know more about recent times than about distant epochs. Now Sikora et al. (2017) have shown that humans from around 34,000 years before present were already organized in small groups with limited within-band kinship and inbreeding, and with wide social, mating networks, resembling the way in which many present-day hunter-gatherers live today. The paper by Sikora et al. is important because of two reasons. First, it shows that certain human behaviors and socializing patterns are quite old, and provided that these are confident proxies for aspects of language design, we are allowed to suggest that humans from that remote period spoke languages close, but not identical, to the first type we described above [what Wray and Grace (2007) call *esoteric languages*]. Second, in their characterization of social dynamics in early Upper Paleolithic, Sikora et al. have not relied on archaeological remains, but on ancient genome sequences. Because protocols for using ancient DNA have been significantly improved over the last years, enabling to reveal snapshots of genetic variation in past populations (Slatkin, 2016; Key et al., 2017), we can anticipate sharp pictures of social dynamics (and accordingly, of language features) from more distant periods of our history, provided that suitable human remains are available. Likewise, knowing more about social dynamics in the remote past, particularly, kinship systems, should help better understand the interrelation between gene and language phylogenies throughout our history (Lansing et al., 2017).

In sum, cutting-edge research in the domain of population genetics and paleogenomics is expected to allow linguists to apply what they have learnt about the languages spoken by present-day human groups and the social conditions favoring their distinctive structural features to a period of our history that is far beyond the limits of the best linguistic reconstructions, and ultimately, to provide with more accurate characterizations of prehistoric languages. Needless to say that caution is in order, particularly, to avoid the temptation of plainly equating modern hunter-gatherers' languages to the languages spoken by prehistoric peoples. No present-day human group is frozen in prehistoric conditions. Change is connatural to language, and subtle modifications of social dynamics and environmental conditions have seemingly occurred over time. The story of click sounds nicely illustrates this: often considered a distinctive feature of the first languages spoken by humans (Knight et al., 2003), linguistic and genetic evidence is also compatible with the view that they might be a recent episode in the diversification of human speech (Güldemann and Stoneking, 2008). That said, and assuming that human cognition has remained substantially the same from the emergence of our species, the patterns of population dynamics and socialization behaviors revealed by paleogenomic studies like the one conducted by Sikora et al. seem a reliable window to the nature of the languages spoken in deep prehistory. These are good news for the fields of historical linguistics and language evolution, but also for anyone interested in human cultural evolution.

## Author contributions

AB-B conceived and wrote the paper. The author confirms being the sole contributor of this work and approved it for publication.

### Conflict of interest statement

The author declares that the research was conducted in the absence of any commercial or financial relationships that could be construed as a potential conflict of interest.
